# All you Need is Trust? Public Perspectives on Consenting to Participate in Genomic Research in the Sri Lankan District of Colombo

**DOI:** 10.1007/s41649-023-00269-y

**Published:** 2023-11-29

**Authors:** Krishani Jayasinghe, W. A. S. Chamika, Kaushalya Jayaweera, Kalpani Abhayasinghe, Lasith Dissanayake, Athula Sumathipala, Jonathan Ives

**Affiliations:** 1 Institute for Research and Development in Health & Social Care, Battaramulla, Sri Lanka; 2 Centre for Ethics in Medicine, Bristol Medical School, University of Bristol, Bristol, UK

**Keywords:** Genomic Medicine, Research ethics, Therapeutic Misconception, Informed Consent, Trust, LMIC

## Abstract

Engagement with genomic medicine and research has increased globally during the past few decades, including rapid developments in Sri Lanka. Genomic research is carried out in Sri Lanka on a variety of scales and with different aims and perspectives. However, there are concerns about participants' understanding of genomic research, including the validity of informed consent. This article reports a qualitative study aiming to explore the understanding, knowledge, and attitudes of the Sri Lankan public towards genomic medicine and to inform the development of an effective and appropriate process for informed consent in that setting. Purposive sampling was employed. Participants were recruited from a sub-group of the public in Colombo, Sri Lanka who had either consented or refused to donate genetic material for a biobank. Data were collected using face-to-face semi-structured interviews. Interview data were transcribed verbatim and translated into English. Conventional content analysis was used. The analysis developed three key themes: a) ‘Scientific literacy’ describes an apparent lack of scientific knowledge that seems to affect a participant’s ability to understand the research, b) ‘Motivation’ describes narratives about why participants chose (not) to take part in the research, despite not understanding it, and c) ‘Trust’ describes how trust served to mitigate the apparent ethical deficit created by not being fully informed. In this article, we argue that informed trust is likely an acceptable basis for consent, particularly in settings where scientific literacy might be low. However, researchers must work to be worthy of that trust and ensure that misconceptions are actively addressed.

## Introduction

Genomic medicine has, arguably, now entered the medical mainstream, and is fast becoming a critical component in medical practice and research, with the expectation that it can provide powerful tools to alleviate human suffering through the prevention, diagnosis, and treatment of disease (Collins and McKusick 2001).

Interest in, and engagement with, genomic medicine and research has increased globally during the past few decades with numerous technological advances and discoveries about the genetic basis of many diseases. The World Health Organisation (WHO) has expressed concern that the advance of genomics will further increase the health gap between developed and developing countries (WHO 2002).

Some literature has documented the minimal involvement of lower-middle-income countries (LMICs) in genomic medicine and research, despite the acknowledgement of its value in these countries (Calsbeek et al. 2007; WHO 2022). Whilst attempts to ‘narrow the genomic gap’ between LMICs and higher income countries are *prima facie* a good thing, rushing ahead to close the gap may create longer-term problems, as genomics needs to be considered in the context of the entire health system and local culture (Calsbeek et al. 2007). A recent survey of LMICs in the Arab region showed that awareness of biobanking is not high (less than 50%), willingness to donate is low (less than 10%), and that respondents “harboured concerns with trust, privacy and data sharing” (Ahram et al 2022).

## Sri Lanka and Genomic Research

Genomic developments are increasing rapidly in Sri Lanka (Sirisena et al. 2016), with genetic and molecular diagnostics introduced around 2001, and local genomic research and development competencies have grown progressively since then (Manamperi and Huzair 2012).

The importance of building capacity for research and development in genomic medicine has been recognised in Sri Lanka, where basic infrastructure and institutional support already exist. At present, there are several state-owned and private institutions engaging in molecular diagnostics and genomic research, on a variety of scales and with different aims and perspectives. For example, the Institute for Research and Development (IRD) is a non-governmental, not-for-profit institution that has established a genetic laboratory and biobank as part of their Colombo Twin and Singleton follow-up study (COTASS-2) (Jayaweera et al. 2018, 2020; Sumathipala et al. 2013). This biobank will be used to undertake epigenetic, genome-wide association, and candidate gene studies to better understand the gene–environment interplay in several medical conditions (Sumathipala et al. 2013). However, recruitment to this bio-bank has seen mixed success, with investigators raising questions about the extent to which potential participants sufficiently understand the potential benefits of genomic research, but also about how this understanding can be developed in a balanced and ethical manner (Sumathipala et al. 2013). These questions boil down to asking how good quality informed consent can be facilitated.

## Informed Consent

Informed consent is the formal process of ensuring that potential research participants understand sufficiently what they are being asked to do so that they can make an informed decision about whether or not to participate. Informed consent is one mechanism through which researchers respect the autonomy of participants and is premised on the idea that persons with capacity have the right to determine the course of their own life (as far as possible), based on sufficient understanding and without being manipulated, deceived, or coerced into any activity at odds with their self-chosen life goals.

Obtaining informed consent for participation in research can be challenging, particularly so for a complex field such as genomics. Traditional approaches to designing informed consent processes rely on ‘experts’ determining what information participants need to know to make an appropriately informed decision, but this model is increasingly viewed as problematic for its failure to be attentive to the needs, values, and beliefs of the communities engaged by the research. Additionally, and from a more pragmatic perspective, since community acceptance and engagement are major factors in the success of genomic research (Calsbeek et al. 2007; Etchegary et al. 2015), attention to community engagement is required to develop appropriate informed consent processes, with improved consent processes being one reason to engage patients and the public in research (Ives et al. 2016).

The literacy, knowledge, and attitudes of potential participants toward genomic research are significant factors in consent (Condit 2010; Jamal et al. 2014; Shabani et al. 2014; Wonkam et al. 2006), and these are likely to vary depending on geographical, cultural, and social differences (Aro et al. 1997; Catz et al. 2005; Haga et al. 2013; Jamal et al. 2014). Consequently, it cannot be assumed that informed consent practices developed in one context will be appropriate for all. Additionally, given that genomic technologies can potentially obtain information about an individual that also provides information about disease and disease risk in family members or specific populations including, for example, tribal communities (De Vries et al. 2011), and donating bodily material can often have particular religious and cultural significance, it is important that genomic research planning, including consent processes, directly involves the *populations* under study (Adlan 2015), and not just the individuals.

There is, therefore, a need to understand the culture, values, beliefs, norms, and attitudes of the specific research population with whom we are seeking to engage, before introducing genomic research. It is only through this engagement that appropriately contextualised informed consent processes can be developed.

## The Sri Lankan Context

There is indirect evidence of negative attitudes toward genetic research in Sri Lanka (Sumathipala et al. 2013). A previous study of public and professional perspectives has highlighted potential limitations in public understanding of 'research' (Sumathipala et al. 2010). Some information about genomic research and medicine in Sri Lanka comes from media reports, which tend to be sensationalised and highlight the exploitation of Sri Lankan genetic heritage through the exportation of genetic material abroad (Sumathipala et al. 2013). Such reports may generate negative attitudes toward genetic research that can impact its viability. For example, the IRD met substantial resistance when founding its genetics laboratory, taking five years for it to be established (Siriwardhana et al. 2011). A small number of studies (Dissanayake et al. 2002; Jayasekara 1989; Jayasekara et al. 1988; Simpson 2014), report positive attitudes towards genomics amongst health professionals in Sri Lanka. These data, however, represent the views of a minority expert group rather than those of the public, whose engagement is key to its success.

The need remains for a more nuanced understanding of what research participants and their communities understand by medical research in general, and genetic research in particular (Kengne-Ouafo et al. 2016), so that information can be tailored and communicated in a way that achieves the goals of obtaining both informed consent and informed refusal.

This research, therefore, aimed to explore the understanding, knowledge, and attitudes of the Sri Lankan public towards genomic medicine which could inform the development of an effective and appropriate process for informed consent in that setting.

Our approach to this study can be situated in the field of empirical bioethics, with a naturalistic commitment to the idea that studying empirically the real world can inform theorising about how the world ought to be (Ives et al. 2016). There are multiple, and complex, methodologies within empirical bioethics, and we adopt a consultative (Davies et al. 2015) methodology, in which we collect and analyse qualitative data about stakeholder’s views and experiences, and consider what that experience can tell us about the way we ought to think about consent in a Sri Lankan context. We adopt a methodology of reflexive balancing (Ives 2014) which involves: (1) identification of a moral problem; (2) empirical enquiry into the problem and the formulation of normative hypotheses; and (3) testing those hypotheses, so they are either defended, rejected or amended. We report only the first two stages here. As such, we outline our qualitative methods and present data that pertains to our subject of interest, followed by a brief discussion that highlights how our data problematises the ‘western ideal’ of informed consent. We then hypothesise that a reorientation of the way we think about informed consent might be appropriate, and justified, in a Sri Lankan context. Given space limitations, we are unable to go further than that in this paper.

## Methods

This study aimed to answer three key questions:

1. What do participants understand about the aims and purpose of genomic medicine and research?
2. What do participants think are the risks and benefits of genomic medicine and research?
3. Do participants think that genomic medicine and research should be promoted and/or supported, and why?

Participants were recruited from a sub-group of the Colombo public who had either consented or refused to donate genetic material for the bio-banking component of the Colombo Twin and Singleton Follow-up Study (COTASS-2) (Jayaweera et al. 2018). The aim was to select a particular subset of the public who have prior experience of engagement with such research and can reflect on that experience, rather than considering it hypothetically.

Purposive sampling was employed to achieve a range of ages and an equal gender split, to access diverse perspectives rather than generalisability. Potential participants were identified from contacts held by the COTASS-2 study (who had previously consented to be contacted) from lists of persons who had consented and refused to participate in the biobank. Individuals were randomly selected from these lists and contacted by telephone in small batches until sufficient numbers had been recruited.

Participants were offered LKR500 (~ USD2.7) as compensation for their time at the end of the interview, which was viewed as appropriate in the local setting. Informed consent was obtained from all participants.

Data were collected using face-to-face semi-structured interviews, comprising open-ended questions, undertaken between October 2017—February 2018. Interviews were carried out by trained research assistants (SC and KA), conducted in Sinhala, and audio recorded.

Interview data were transcribed verbatim and translated into English. Conventional content analysis was used (Hsieh and Shannon 2005), wherein the text was initially coded for content relevant to the research questions, and then grouped into family codes. Both descriptive and interpretive themes were developed by exploring the relationships between the codes and family codes, with constant comparison and deviant case analysis employed (Silverman 2017), to add rigour. A form of analytic triangulation was employed, by which the initial coding was performed by KrJ, which was then checked by JI and AS. Finally, all authors agreed on the final themes. Coding was carried out on English transcripts, as this was the common language among all researchers, but as the analysis progressed key passages were periodically back-translated in Sinhala and the meaning double checked, to ensure that we remained true to participants’ voices. Regular analysis meetings were held between KrJ and JI, during which developing themes were discussed and agreed upon. Whilst none of these practices will ensure the ‘correctness’ of the findings, they help facilitate the trustworthiness and credibility of the analysis.

## Results

The demographic characteristics of the 10 participants are in Table 1.

**Table 1:** Demographic information of participants

Pseudonym	Age	Gender	Marital status	Highest Education Level	Religion	Employment status
Ahmed	24	Male	Never married	Passed O/Ls	Islam	Employed
Kamal	44	Male	Married	Grade 6-O/Ls	Christian	Employed
Amal	44	Male	Married	Grade 1–5	Buddhist	Employed
Jayathissa	62	Male	Married	Grade 6-O/Ls	Buddhist	Non-employed
Bimali	21	Female	Never married	Up to /passed A/Ls	Buddhist	Non-employed
Saumya	41	Female	Married	Grade 6-O/Ls	Buddhist	Non-employed
Gayani	44	Female	Separated	Grade 6-O/Ls	Buddhist	Employed
Fazna	56	Female	Married	Grade 6-O/Ls	Islam	Non-employed
Nandawathi	80	Female	Widowed	Grade 6-O/Ls	Buddhist	Non-employed
Aleema	40	Female	Married	Up to /passed A/Ls	Islam	Employed

Our analysis developed three key themes. The first two are primarily descriptive. ‘Scientific literacy’ describes an apparent lack of scientific knowledge that seems to affect a participant’s ability to understand the research. This is compounded by an incomplete understanding of what genomic medicine and research are, therapeutic misconception, and, for some, inaccurate assumptions about who performs the research. The theme ‘Motivation’ then describes narratives about why participants chose (not) to take part in the research, despite not understanding it. The findings suggest a motivation driven mainly by altruism and a feeling of obligation to contribute. However, this is often combined with an expectation they will personally benefit (the therapeutic misconception).

These themes prompted us to ask whether the combination of poor scientific literacy and motivational factors led to participants not providing properly informed consent/refusal. We interrogated the data for content that may shed light on this and developed the theme of ‘Trust’ which, in participant narratives, served to mitigate the apparent ethical deficit created by not being fully informed. Our data suggest that trust plays a crucial role in explaining how people can feel comfortable participating in research, in the context of a participant population motivated by altruism but harbouring persistent misconceptions.

## Scientific Literacy

Participants’ descriptions of the biobank study they had experience of, and of research in general, suggests poor or incomplete understanding – which would, *prima facie*, fall below the level required for informed consent. The gaps in understanding were more to do with fundamental scientific literacy than a failure to recall details about the biobank specifically (memory of which is likely to fade over time). We illustrate this finding via two sub-themes: ‘Gaps in Understanding’ and ‘Therapeutic Misconception’.

### Gaps in Understanding

When asked if they could explain what genomic research is, and how their samples were being used, only a few participants could articulate even a cursory understanding. For example, Ahmed, who consented to take part in the biobank, thought DNA tests were used to check for serious disease and genomic research was about confirming familial relationships—attributing this knowledge to the movies:

*Ahmed: Genes mean, normally in the movies they say about DNA tests. From that, usually, if there is any serious problem, they do check-ups related to DNA. (…) things like that. [Genetic research] means normally mm… whether some individuals are members of the same family can be identified through a DNA test. I have a rough idea like that.*

Fazna gave a similar account, saying that the purpose and role of genetic science was to prove familial relationships:

*Fazna: Now this is my son, you see… even though I refuse it can be proved by doing a genetic test… And also, you can identify this is my son and I am his mother… that’s all I know… From watching television…reading newspapers…social knowledge.*

The majority said their main information sources were television, newspapers, and social media:

*Aleema: I watch the news and read papers usually …I watch videos on the internet… and thereby I had some basic details.*

There was some understanding of the possible risks of genetic research, both personal and general. For example, Amal felt there were risks for both individuals and society:

*Amal: I would look at the research if it doesn’t carry any risk for own self… a… a… and if it does a job either to the country or to the society or an individual, supporting that is what should happen. …a risk to own self means… Those genetic… say it’s my materials… from the materials collected from me… Say I have something in some way. Like a disease… imagine I have a disease… then I will have to think and proceed with respect to the disease… saying that I’ve got a disease… I should get treatment for this… that this treatment method should be used… Likewise, a person might become depressed over those things or might tolerate… that could vary from person to person.*

Some participants expressed worries about what genetic science can do in general. For example, Ahmed articulated concern about promoting genetic research, and was worried about it being used to create human beings:

*Ahmed: I mean… I don’t know much about that… Normally… another human being can be created by using another person’s DNA, I mean… I have heard something like that, and therefore I think those are not good things.*

Whilst some were able to express specific concerns about possible risks, few demonstrated a clear understanding of potential benefits. Rather, they tended to simply assert that it must be beneficial:

*Amal: …there has to be a benefit if medicine is concerned. Unless there’s no reason to conduct these things… It’s hard to describe that in different aspects…. I don’t have knowledge or an understanding … It’s difficult for me to explain gene technology in simple… there has to be an understanding to do that… otherwise, cannot explain, right?*

However, some participants talked about the genetic transmission of illness, and how some diseases/illnesses can be passed on to children from their parents:

*Gayani: Now if they have things like diabetes and cholesterol those also can be transmitted. When you go to the doctor, they ask whether your mother has these diseases and your father has these diseases. They ask it because of this fact. That’s why.*

Overall, participants tended to get their information about genomic medicine and research from television and other media and associated it with parental testing. Participants ﻿tended to think there were benefits and risks, but could not generally articulate them and these were often based on misunderstanding. Participants frequently acknowledged their lack of understanding and often requested clarification.

### Therapeutic Misconception

One very specific area of misunderstanding that became evident, related specifically to participation in COTASS-II, was confusion between research and therapy. Some participants reported taking part because they thought it would bring free medical check-ups and diagnoses.

For example, Gayani implied that she expected to be told about any possible health issues identified from her sample. When asked directly, she confirmed as follows:

*R: Then miss, do you hope that they will inform you about the new findings?*

*Gayani: I hope they will. Yes… About what are the diseases we have and what we don’t, right?… I will be happy if they inform me like that.*

For context, it is important to note that on the information sheet this participant was given it was clearly stated that no information obtained from the genetic sample would be fed back to them.

Generally, participants wrongly associated genomic research with diagnostic testing, thinking that their samples were being tested for personal health issues and expected they would be informed if their sample indicated disease. For example, when asked to explain what genetic research is, Jayathissa said that it was about “*blood tests and all done in the body*”, with the aim of checking “*if there is diabetes, other diseases this and that…. then cholesterol, such things*”. A follow-up question asked why he would take part in genetic research, and his reply was indicative of the therapeutic misconception:

*Jayathissa: I will join. Yes… with the intention of finding out whether I also have any diseases or illnesses.*

The therapeutic misconception was also evident in the way some participants talked about whether people should take part in genomic research – suggesting that you would only need to participate if you have a problem that needs investigation:

*Ahmed: Generally, I mean… if they have any problem or a problem about the details of their family it is ok to [participate], otherwise, I don’t think it is necessary.*

This therapeutic misconception may be related to the fact that some participants tended to believe genetic research was carried out by doctors, whom they associate with therapy:

*Supan: Generally, mm… It’s doctors who mainly do genetic research. It is them who take and check the DNA of individuals and find the required information.*

*Kamal: Someone from the hospital, not from a company… It should be some hospital anyhow, right? Otherwise, it can’t be just a company, I think it’s at some hospital.*

Other participants believed that genetic research is carried out by a mixture of clinicians and researchers, including research students:

*Aleema: so, it could be doctors…or else students in research institutes…. if not, those who are studying further in these subjects…*

*Amal: Usually, specialist doctors and scientists might be doing some discoveries…*

## Motivation

Overall participants tended to describe their main motivation as altruistic, believing their participation would benefit others.

The anticipated benefits varied, as did anticipated beneficiaries. A good illustration of this comes from Aleema, who describes her motivation in terms of wanting to serve her country, bringing benefit to ‘someone’, but also to benefit the individual researcher:

*Aleema: why not, it is a service to the country… so, I thought it would help someone in any way… so I liked it and said I will go through that. …to be frank, I gave the consent because you are [the researcher] benefited by this… to tell the truth I don’t have any advantage from this according to my opinion…*

Other participants had less mixed motivations, with a clear expectation that in participating they would benefit others on a large scale. They believed research is intrinsically good and intended to help people:

*Saumya: Yes, I like it, if it serves the public in some way… that is what I want. …. Anyway, I have diabetes. have cholesterol. Therefore, I know the suffering I endure… thus, I like it if some of that blood is taken, and more is found about them. if more findings are generated, if more good things happen… isn’t that a great thing… It would make me happy if through me there is some service to the public.*

*Amal: if it is done successfully, it definitely should do a favour for the existence of humans… I feel it would be a great service for society, and human lives if something is done through that… Genetically transmitted problems are common to both humans or animals, aren’t they? Every living being has genetic components, right? So, some good happens to humans by doing these things through different discoveries… …it doesn’t matter wherever [it goes] if it benefits humans…*

Related to the question of motivation, and benefit, was the question of ownership. In the context of research in LMIC settings this is particularly important, given ongoing concerns about exploitation and benefit sharing. Whilst participants generally spoke in altruistic terms, there was often a caveat that the benefit must be accessible to them and their society.

In terms of simple ownership, some participants felt they owned their samples and allow them to be used to benefit others. For some, that ownership meant that they can and should also benefit personally.

*Aleema: we own our sample anyway… we give on their request…but we have the ownership…*

*Amal: they are my stuff and it’s [more] important to me than anyone else does… Despite the fact that you’ve taken and regardless of its good or bad outcomes, it’s complete ownership and right to know should be for the participants … they are the people in need to know, anything of it should be for them. Therefore, the ownership should be for them… one who takes it is taken for the research, right? If there is any outcome of that particular research, it is not for him… but for the patient… one who has taken it and experienced it. Therefore, the right is for the patient.*

Others felt that the sample was simply owned by the researchers, whereas some qualified that ownership as partial and dependent on it being used to benefit of others:

*R: If so, Now, they take a sample from you for research, who has the ownership of that sample?*

*Bimali: To the medical research institute… To the people who have that sample*

*Fazna: It belongs to me… I cannot fight with them or force them to say it belongs to me… they took it for a purpose… therefore it belongs to me… although it belongs to me it is necessary for the research, therefore (3/4) of it belongs to them and (1/4) of it belongs to me… Therefore, I cannot fight here saying it belongs to me… my blood… it’s mine… it was done using my genes, I cannot keep saying these. …but they own it more.*

Participants in general tended to be happy to give up ownership claims or control over their sample, in the expectation of others benefitting. They did, however, also tend to feel that they should personally be in a position to share those benefits and that the benefits that arise from their contribution should be distributed fairly. For Gayani, this included giving people free access to any health benefits arising from their contribution. When asked how she would feel if a benefit was not shared with her, but sold, she responded as follows:

*Gayani: That’s not fair, isn’t it?… It’s like they have drawn our blood and sold it. Finding diseases from us. Finding cures for those. I don’t like selling like that. It’s better if all are being benefitted.*

When asked a similar question, Bimali and Saumya did not feel that free access was required, only that the benefits should be made available to the Sri Lankan people and beyond:

*Bimali: We don’t need it freely or for a less price. If it's useful to society as a whole, it's no good to sell to a foreign country.*

*Saumya: We are all human beings… not only to this village it should be provided to the whole of Sri Lanka… It should be provided to the whole world, not only to one person.*

## Trust

The results described thus far paint a picture of a population who have either consented, or withheld consent, to provide a genetic sample for biobank research, but whose understanding of the research suggests that consent might not be sufficiently informed. In particular, we have presented examples of relatively poor understanding of what genetic research is and what its benefits and risks are, and we have clear evidence of therapeutic misconception. However, we can also see that many participants were aware of the limits of their understanding and happy to consent regardless.

We have presented examples that demonstrate participation can be motivated by a sense of altruism and the expectation of wider benefits being shared, but coupled with an inability to articulate what those benefits are. This leads us to question whether the consent that these participants gave (or withheld) was sufficiently informed, and why participants may have chosen to take part despite being aware they did not fully understand what the research was doing.

On interrogating the data with this question in mind, it became apparent that ‘trust’ was doing a great deal of work. For example, the decision to give consent was taken by some participants because, even though they did not fully understand the research, they trusted the person who was asking for consent. Further, participants felt able to act on their motivation to benefit others because they trusted the research would bring benefit – even if they did not fully understand what that benefit would be. Participants were willing to relinquish ownership and control of their samples because they felt they could trust the researcher to use their samples for the benefit of others and to share those benefits. We will illustrate this theme with some examples.

First, it is important to note that during data collection, there was a general sense of scepticism about research, generated by reports that criminals in the Colombo area were entering peoples’ homes posing as researchers. This may partially explain why the issue of identity verification came up. A pre-requisite for trust appeared to be the ability to verify that the person they are speaking to is *bone fide.* The following quotation from Saumya illustrates this thought process well:

*Saumya: if an ordinary person or someone comes and asks for a such test, we wouldn’t know who that is, we have to participate only if he can prove his identity accurately in writing, in general, to do something like that it has to be one of the health departments, right? or else, a person who walks by this road can’t just come and do it, can he? We need such verification or something, there has to be a certificate.*

Ahmed and Aleema also highlight the importance of identity checking to verify trustworthiness:

*Ahmed: They should have some identity cards. I will check that.*

*Aleema: whether they are from a legalized place… whether they have the approval of the medical council…what they are going to collect and in which method… either we can check their identity… or their approval… mainly from that.*

This verification was required for the conversation to begin and was a necessary condition of trust, but not sufficient. For many participants, the decision to participate was based on whether they felt they could trust the person taking consent, based on the way they conducted themselves and the reassurances given.

For example, Fazna talked about harbouring a general sense of distrust and expressed concern about fraud but then, talking about *this* research rather than the biobank study, she explained she was talking to our interviewer right now only because she had decided he was trustworthy:

*Fazna: Nowadays society is 25% truth and 75% lies… therefore although you showed me all your legal documents, I don’t believe it 100%. I don’t want to get deceived. My sister also told me the same thing. We are not sure what this is all about. Why do you want our blood samples? will they come again to take our blood samples saying that our blood is ideal for the research. But this is only for the research… this is good if it’s not a fraud… …I trust you by the way you talk now… … I understand you… this is not a fraud… I trust this research and I admit that this is good.*

Talking more generally about participation in the biobank research, Ahmed expressed the view that he would be willing to accept reassurance from the researcher at face value:

*Ahmed: …I can say this, eh… I can participate in your tests. But eh… I can do it if you can guarantee that I won’t have any trouble… if you can confirm then I don’t have any problem in the future, mm… I don’t mind that…*

Similarly, when asked about future uses of samples, Saumya insisted she did not need to know the details – she only needed to trust.

*Saumya: believing that you all are genuine, I made that assumption… that means building trust, that is what you need to have… …Now, let’s say that you took a sample… now, another individual comes… he is also provided with it…. then it would not be known if it the same thing that is occurring… That is the problem that exists… If it is that same thing that is happening, I don’t have a problem… Only trust is required.*

Overall, participants tended to recognise and accept their understanding was partial, and felt their knowledge about future uses of the samples could only be partial. Recognising that gap, participants did not seem concerned about filling it. Rather, they sought to *bridge* it by deciding to trust the researcher and, vicariously, the research institution.

When asked about what is required to enable them to trust, some participants recalled their belief that research is usually conducted by doctors and that doctors were trustworthy. Having doctors on the research team seemed, therefore, to imply trustworthiness:

*Amal…the one who’s accepted in society is the doctor, right? Even during illnesses, we first go looking for a doctor. Since this research is also led by a team of doctors, I would support this without hesitance…*

*Fazna: there can’t be a risk, they don’t kill people. doctors neither, doctors are there to save people or to keep them alive for at least an hour. or else they don’t kill people*

Another indicator of trustworthiness, specific to COTASS-II, was knowing the organisation, with Ahmed and Saumya attributing their act of trusting to knowing the IRD and/or the Sri Lankan Twin Registry:

*Ahmed: …Mm… considering this twin association, generally, it is famous. I will give this due to the trust I have in you*

*Saumya: …that twin organization, has existed for many years…. Therefore, I thought that this was something genuine… when I was told that this is private, I felt a little uncertain… however, I also felt that it is genuine… as you have been providing your service for several years…*

Similarly, association with government and/or ‘official’ research was stated to enhance trustworthiness – according to Kamal because it guarantees the research is properly motivated.

*Kamal: Well, it should be done by some government thing. People like it if done by some state thing. As in, it has recognition then. We wouldn’t know what it is for if done by some private thing. There’s no guarantee whether this was taken to do any huge business. A lot of people would prefer if anything is done through the government.*

Saumya made clear she felt private organisations can also be trusted although, interestingly, she required clarification about whether the COTASS-II study was government-run or private, before confirming that she had decided to trust.

*Saumya: Now, is this being conducted by the government… or else…I don’t mind it being private… trust is what needs to be protected…. (Participant asked the researcher whether this is a government organization or a private one; the researcher confirmed it is private) … I provided this to you based on trust… because I believe that here you will not do such a thing…*

For some participants, the decision to participate was influenced by the attitude of family or close others. For example, Nandawathi explained how her husband’s attitude toward the research motivated her to participate:

*Nandawathi: As for a reason… my husband was there that time, I told him, “You go and participate”, then he said, “no no, you should be participating”. So, I got involved.*

Saumya reported that another participant influenced her decision to trust the COTASS-II researcher:

*Saumya: I informed my elder brother… my elder brother isn’t always at home… he goes for a job… so there is another daughter of an aunt…. She is also a twin… she is also on this road… the last time (COTASS-2), I met her on the road… She asked me whether the twin’s organization visited… I said that they came… therefore from that as well I developed trust…*

Amal reported seeking advice from her GP, who verified aspects of the study that enabled the trust to be given:

*Amal: I encountered a doctor and inquired whether there’s a programme like this and what they do from that. Then that doctor said such a programme is being conducted using Sri Lankan materials. So, he said that… this is done along with the approval of the government, but he’s unable to say about the results, but he confirmed that such a program was going on so I accepted then. If a doctor who didn’t know all about this said so I had no necessity to disagree or doubt it, right?*

Another factor reported to facilitate trust was information provision. This seems initially at odds with the previously reported finding that information is often not fully understood. However, in this context, we can hypothesise that the act of providing information, even though it may not be understood, is demonstrative of transparency. That in itself seems sufficient to reassure participants there is nothing to hide. It demonstrates trustworthiness because they have been provided with information that they can,﻿ if wanted, verify. For example, Nandawathi described how receiving information beforehand allowed her to feel safe:

*Nandawathi: We have to be informed and after getting to know only we should participate, otherwise we don’t know who that person is, we can’t say if he’s a thief or not, we don’t know which kind of people are coming with the current situation…You came here after sending a letter and informing us beforehand, so I knew that you were coming for this reason, so I participated, if another comes, I wouldn’t join. I will refuse. We can’t get involved without knowing, who knows for what reasons they come, never know that they are to murder, that’s the situation today.).*

Gayani and Fazna talked about the importance of having information about what samples are being taken and what is being investigated, but still focussed on having enough information about the people conducting it and the status of the organisation. This suggests that the latter – what Jayathissa calls “the essential stuff” is more important to them than understanding what the research involves:

*Fazna: I cannot give my approval without knowing what is the purpose and whether will it be beneficial to others… Since various kind of people is approaching, that’s it…Only after investigating the institute. Cannot give without investigating the institute.*

*Gayani: Now firstly we must ask whether they are taking blood or skin samples… We cannot allow it right away, right?*

*R: Yes, ok then miss, will you change your decision on joining it according to what they are taking from you?*

*Gayani: We cannot allow them to get only what they want, right? We should also observe. They will be allowed to take samples after asking them about the details. Cannot give without investigating about the institute… through the telephone or from addresses you can investigate about them…*

*Jayathissa: if someone comes from an institute… there’s a name for that institute also… so, you need to confirm that, right… So, I will inquire them what they are doing … We will participate only after we get to know the essential stuff.*

Another factor, related to the facilitation of trust and transparency, was the preference of some participants for verbal, over written, information. The latter was not felt to be adequate. Literacy is high in this population, and so we view this as less about struggling to digest written information per se and more about the valuing of human interaction as a means of allowing participants to determine whether they can trust ‘this person’ and therefore the research they represent. Making people available to answer questions is a form of transparency that invites trust, and this cannot be achieved with an information sheet and a one-off consent meeting – as described by Amal and Saumya:

*Amal: talking face-to-face will build trust and will be successful too. And clearer than when someone is talking over the phone. I think it is better and more fruitful than reading a sheet or talking on the phone. I think a discussion done in that way is more successful than doing it without seeing.*

*Saumya: you should at least explain over the phone… that is the most important thing… After looking at this certain people simply ignore it, believe that it is of no relevance… Certain people… Even I felt like doing it… this was relevant to me because I am suffering from ailments… Because I am unwell, I take pity on other people… I decided that I shouldn’t ignore it… That is why every time you called, I requested for a time to be given… so that time could be allocated for this… I felt that, if this gets left behind it would be a waste… therefore I think explaining… If the explanation was provided over the phone, it would have been much better… Therefore, when you call explain… That was what was most important to me.*

*R: It was after this that you ask for further information to be sent to you.*

*Saumya: Yes yes… you said that it will be sent by mail… likewise, after two or three days it arrived. After that, I trusted this more…*

*R: Then, when providing information for this… about our research…*

*Saumya: Before that, they didn’t approach that way… their interest was established… they came, discussed, and left… therefore, from this more trust was developed…*

## Discussion

The data presented shows that, in a very real sense, people are taking part in genomic research without being *fully* informed. Furthermore, there is clear evidence of misunderstanding, suggesting that consent could not have been informed and is therefore invalid.

Dominant, and predominantly Western, thinking on informed consent would suggest that the consenting in the COTASS-II process was therefore flawed and unethical. People were very likely recruited into the biobank study when they did not have a full understanding of the research, its risks, and benefits, or how the results would be used.

This is not inconsistent with what we already know. Previous studies have shown that, post-involvement, research participants will often have a poor recall, and demonstrate a relatively poor understanding of the research (Bukini et al. 2020; Robinson et al. 2013). This does not, of course, necessarily indicate a sub-par informed consent process, but may merely represent waning recall. What matters for valid consent is what participants understand at the time, and not what they remember 6–12 months afterwards.

What differs here is that some participants seem to describe providing consent in the knowledge that they did not have a full understanding of the research, whilst others describe going in with clear (though not, of course, to them) misapprehensions about how they will benefit. This seems not only to fall below the standard for informed consent but also for understood consent (Isles 2013; Vasquez 2017).

Trust, however, appears to act as a mediator, allowing consent to be given in the absence of full understanding. The idea that trust is an important factor is consistent with the findings of Ahram et al (2022), who associated lack of trust with a low willingness to participate in biobanking. This is also consistent with the characterisation of informed consent articulated by Manson and O’Neill (2007), which holds that due to the imperfection of communication, the notion of *fully informed* consent is unattainable. In the absence of full understanding, informed consent becomes a question of understanding enough to be able to decide to waive the usual social rules that govern behaviour and allow somebody to act upon you.

This, of course, requires trust, and our study participants recognised this, describing a consenting process where they focussed more on making an informed decision about trusting the researcher (and the research) rather than understanding the research. This still seems like an informed decision, albeit with a focus on the human rather than the science. This does not mean that efforts should not be made to provide information to potential participants. It does mean that we can consider ‘informed consent’ to allow for different understandings of what it means to be informed. So long as the decision to trust is itself informed, and that researchers are worthy of that trust, we see no reason to view consent informed by trust as any less valid than consent informed by understanding. This does place the onus on researchers to ensure that they are demonstrably trustworthy, and on institutions and regulators to act against practices they might exploit trusting behaviour.

Whilst an informed decision to trust can, in our view, mean that consent is valid notwithstanding incomplete understanding, there is a difference between entering into research with missing information and with erroneous understanding. We view the therapeutic misconception as an example of the latter, and it is important that participants do not enter into research falsely believing they will personally benefit, as this is both an insult to autonomy and will erode trust in the long term.

It is not clear to what extent our empirical findings can be transferred to similar settings outside of, or even within, Sri Lanka. The question matters because it is important to consider whether these findings, particularly about the use of trust as a mechanism for consent, can be assumed to be true of other settings, or if they are likely to be unique to the participating group. This has implications for whether we might want to propose using informed trust as a basis for consent only in the study setting, for Sri Lankans in general, or across other settings that are sufficiently similar. We have no reason to think there is anything specific or unique about our small group of participants, or about Sri Lankan culture in general, that is likely to make them focus on trust in a way that other groups will not; and it seems to us likely that trust may serve this bridging function in other contexts where there is similar goodwill towards research but a lack of understanding. Care must always be taken, however, not to unreflectively generalise. Our data, and our *empirical* hypothesis about trust being used to bridge the gap in understanding, might be usefully used as a lens to explore and understand other contexts, but it cannot be simplistically assumed to apply.

We have, however (and in keeping with a reflexive balancing approach), used the data to inform a hypothesis about the ethical acceptability of informed trust as a basis for consent. This hypothesis, though informed by our empirical analysis, is not tied to it. As such, that hypothesis might be taken and explored in various different contexts, tested, and either rejected or accepted in further work, which will determine its ethical acceptability in specific contexts. As such, this paper has reported the first two stages of Ives’ (2014) reflexive balancing methodology, with the third yet to be reported.

Our takeaway message from *this* study, then, is that informed trust may be an acceptable basis for consent, particularly in settings where scientific literacy might be low. However, researchers must work to be worthy of that trust and work to ensure that misconceptions are actively addressed.

Our aim in this paper has not been to provide a knock-down argument to establish that informed trust is a sufficient basis for consent, such that it can and should replace informed consent. Rather, we have demonstrated how an in-depth exploration of the experience of a group Sri Lankan people has cast doubt on the appropriateness of importing a Western ideal of informed consent into a context where scientific literacy is low; and proffered a possible solution – informed trust—that (*prima facie*) allows us to reconcile the importance of consent in itself with a context in which it seems unlikely to be achievable (at least, as things currently stand).

Further work will need to be done – reflecting the third stage of reflexive balancing—to explore more fully the normative implications of this suggestion, and as such we have only presented part of the work here. Future work will need to consider (*inter alia)*: how researchers can earn and demonstrate trustworthiness (in a way that does not simply exploit existing trust heuristics); where the balance lies between accepting informed trust and attempting to educate and scientifically inform; and, indeed, whether that latter question itself is premised on a problematic and colonial assumption that scientifically informed consent is the ideal we ought to be striving towards. There may, in fact, be room to explore arguments that informed trust is a superior – and perhaps in some cases more honest – basis for consent, given that being fully informed may never really be achieved – which is certainly in keeping with Manson and O’Neill’s (2007) contention that consent transactions can rarely (if ever) involve full understanding (even in the best of circumstances). Going further, it might also be considered whether informed trust is a more inclusive basis for consent, given that the requirement for a certain level of understanding in order to participate in research could serve to exclude groups of people who cannot demonstrate the required level. This kind of argument would then require consideration of how to ensure trustworthiness in the researcher, and could be explored from various theoretical perspectives – one being to develop a concept of ‘researcher virtue/integrity’ as proposed, for example, by Banks (2018) and Daku (2018).

We do not have space to consider these arguments here but we hope, in this paper, to have contributed to the large and multifaceted debate on consent for research by proposing ‘informed trust’ as a model that may be appropriate in some contexts.
